# A rare case of fatal delayed aortic rupture caused by repeated microtrauma from a posterior rib fracture

**DOI:** 10.1007/s10140-026-02524-9

**Published:** 2026-07-28

**Authors:** Shintaro Isa, Homare Kaga, Ryohei Kuroda, Atsushi Yamada, Shuuji Namiki, Yoto Oh, Yohsuke Makino, Toshitaka Yoshii, Kana Unuma

**Affiliations:** 1 Department of Forensic Medicine, Graduate School of Medical and Dental Sciences, Institute of Science Tokyo, 1-5-45 Yushima, Bunkyo-Ku, Tokyo, ST 113-8519 Japan; 2 Department of Forensic Dentistry, Graduate School of Medical and Dental Sciences, Institute of Science Tokyo, Tokyo, Japan; 3 Department of Orthopaedic and Trauma Research, Graduate School of Medical and Dental Sciences, Institute of Science Tokyo, Tokyo, Japan; 4 Department of Forensic Medicine, Graduate School of Medicine, The University of Tokyo, Tokyo, Japan; 5 Department of Orthopaedic and Spinal Surgery, Graduate School of Medical and Dental Sciences, Institute of Science Tokyo, Tokyo, Japan

**Keywords:** Rib fracture, Delayed aortic injury, Repetitive aortic injury, Hemothorax, Forensic autopsy

## Abstract

Sharp bone fragments can cause damage to major blood vessels or internal organs during the subacute or chronic phases, even if such bone fracture itself does not immediately affect hemodynamics or the patient's condition. Herein, we report a fatal case of delayed aortic rupture in which the anatomically significant relationship between a posterior 8th rib fracture and descending aortic injury was initially overlooked. The patient initially appeared hemodynamically stable under conservative management for fall-related injuries; however, he suddenly collapsed on hospitalization day 10 and subsequently died. Autopsy revealed a full-thickness perforation of the descending thoracic aorta due to the sharp edge of the fractured rib. Retrospective review of premortem computed tomography demonstrated that the rib fracture fragment was extremely close to the aortic wall; however, this finding did not receive sufficient clinical attention. Histologically, the aorta exhibited partially organized adventitial injury, providing objective evidence of repeated premortem contact between the rib fragment and the aortic wall. These findings strongly support delayed traumatic aortic rupture secondary to rib fracture, rather than spontaneous vascular disease. Posterior left lower rib fractures can precipitate delayed and fatal aortic injury. Following initial screening for life-threatening conditions, rib fractures are often managed conservatively, with only pain control and respiratory support. However, posterior left lower rib fractures may warrant consideration for surgical intervention, both for emergent and even prophylactic purposes. This case provides unique pathological and clinical insights into a rare but lethal complication of blunt thoracic trauma, expanding current understanding of delayed aortic injury due to posterior rib fractures.

**Graphical abstract:**

**Figure:**
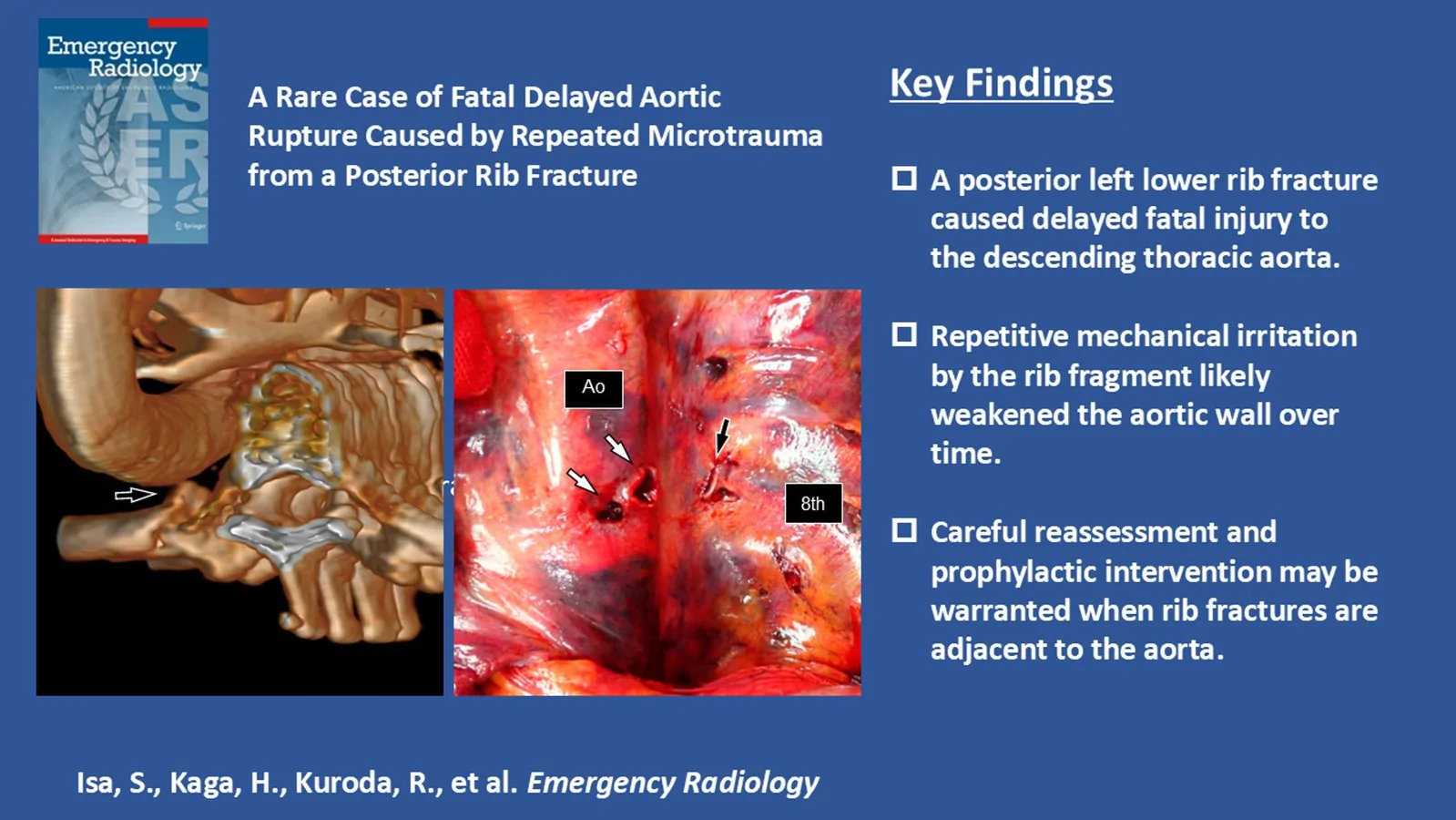

## Introduction

Rib fractures are common in blunt thoracic trauma and are often associated with complications such as hemothorax, pneumothorax, flail chest, and solid organ injury [1–5]. Although most cases are treated conservatively, posterior rib fractures can rarely cause delayed aortic injury when a sharp fracture edge penetrates the thoracic aorta, leading to sudden massive hemothorax days after trauma [6, 7]. Fractures of the left posterior 4th–10th ribs are particularly dangerous because of their close anatomical relationship to the descending thoracic and upper abdominal aorta [8, 9]. In patients with multiple rib fractures or flail chest, minor physiological movements, including respiration, posture changes, or slight trunk rotation, may repeatedly irritate the aortic wall, resulting in progressive vascular injury and eventual catastrophic rupture [10–13]. A forensic autopsy case of delayed traumatic aortic laceration has also been reported [14]. Herein, we report a fatal case of delayed aortic injury following posterior left rib fractures caused by a fall from height, highlighting the progressive mechanism of delayed traumatic aortic rupture from a forensic perspective.

## Case presentation

### Case history

**Fig. 1:**
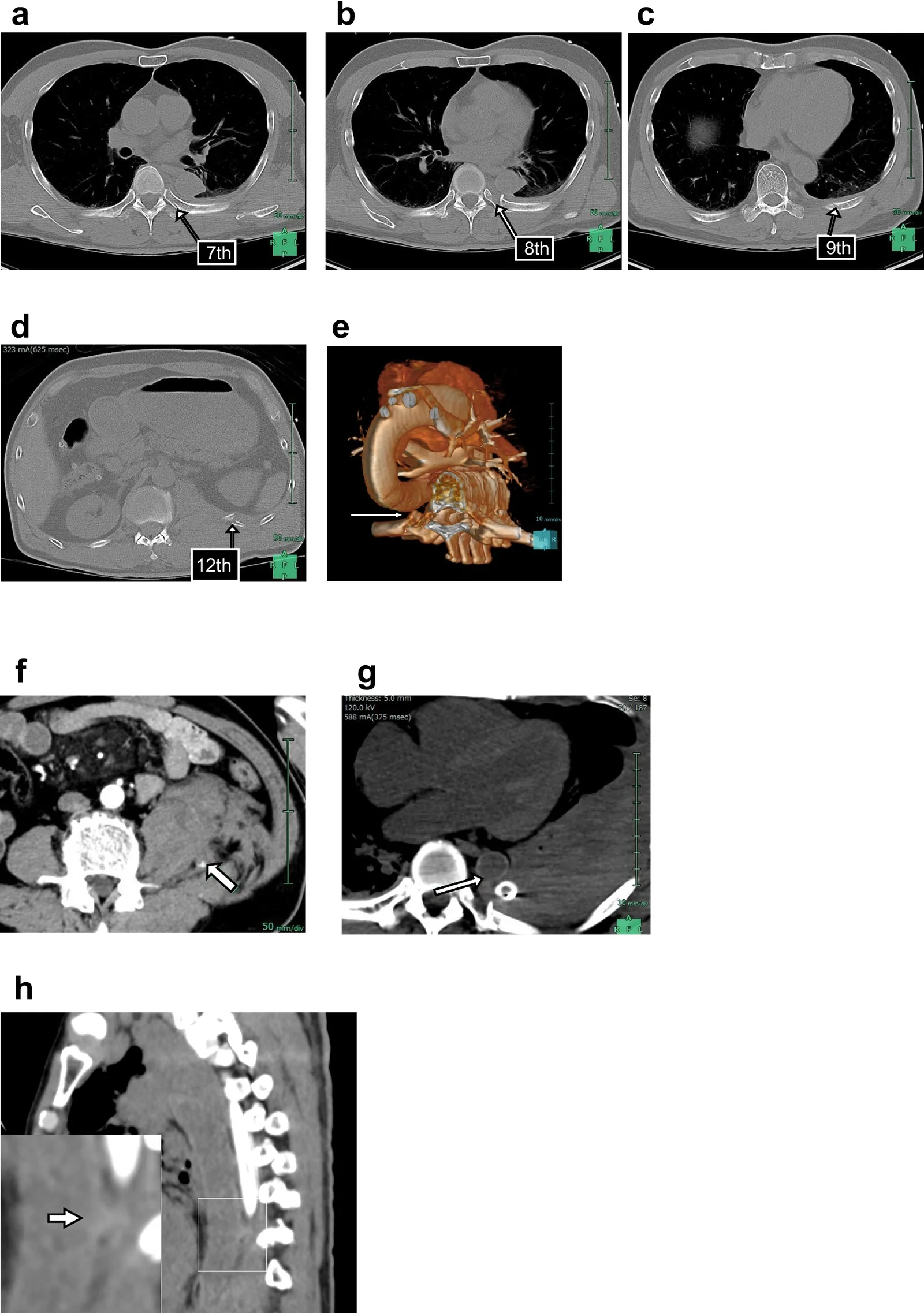
Initial CT images performed on day 1. CT images reveal fractures in (**a**) the left seventh, (**b**) eighth, (**c**) ninth and (**d**) twelfth posterior ribs (arrows) with a small amount of hemothorax. Image with the eighth rib fracture (**b**) reveals internal cortical bone of the fracture site oriented anteriorly, adjacent to the thoracic descending aorta (arrow). (**e**) 3D reconstruction shows the proximity (arrow). CT images of the upper abdomen depict swelling of the left iliopsoas muscle and a suspected pseudoaneurysm of the left L3 lumbar artery (**f**) with retroperitoneal hematoma. (**g**) Axial and (**h**) sagittal post-mortem computed tomography images reveal aortic wall irregularity, suggesting a rupture site. CT, computed tomography

On day 1, a carpenter man in his 60s fell on his back from a roof (height: 4.6 m) and was transported to the emergency department. He presented with lower back pain but remained hemodynamically stable. Initial contrast-enhanced computed tomography (CT) revealed left posterior rib fractures (7th–12th) (Fig. 1a–e), minimal left hemothorax without extravasation, a left scapular fracture, and a small hypodensity in the left Sylvian fissure. A retroperitoneal hematoma with a suspected lumbar artery pseudoaneurysm was observed (Fig. 1f). Repeat CT performed three hours later revealed retroperitoneal hematoma enlargement, prompting transfer to a tertiary hospital on the same day.

At the tertiary hospital, angiography was performed for the suspected lumbar artery injury. Thereafter, conservative management and routine monitoring were initiated. Subsequent CT demonstrated an increased left pleural fluid volume. No dedicated follow-up chest CT was performed thereafter. However, the lower thorax was included on a non-contrast abdominal CT on day 7, demonstrating persistent left pleural effusion with slightly high attenuation (mean, approximately 30 HU). Once retroperitoneal bleeding was deemed adequately controlled, the patient was transferred back to the referring hospital.

On day 10 of hospitalization, while eating lunch in an upright seated position, the patient suddenly became hypotensive and unresponsive. Resuscitative endovascular balloon occlusion of the aorta was performed for suspected recurrent retroperitoneal hemorrhage. Eventually, ultrasonography revealed a large left pleural effusion, and chest drainage yielded 1500 mL of hemorrhagic fluid. Although spontaneous circulation returned transiently, the patient died shortly thereafter. A post-mortem CT at the hospital was obtained (Fig. 1g,h), and a judicial autopsy was performed nine days later.

### Autopsy findings

**Fig. 2:**
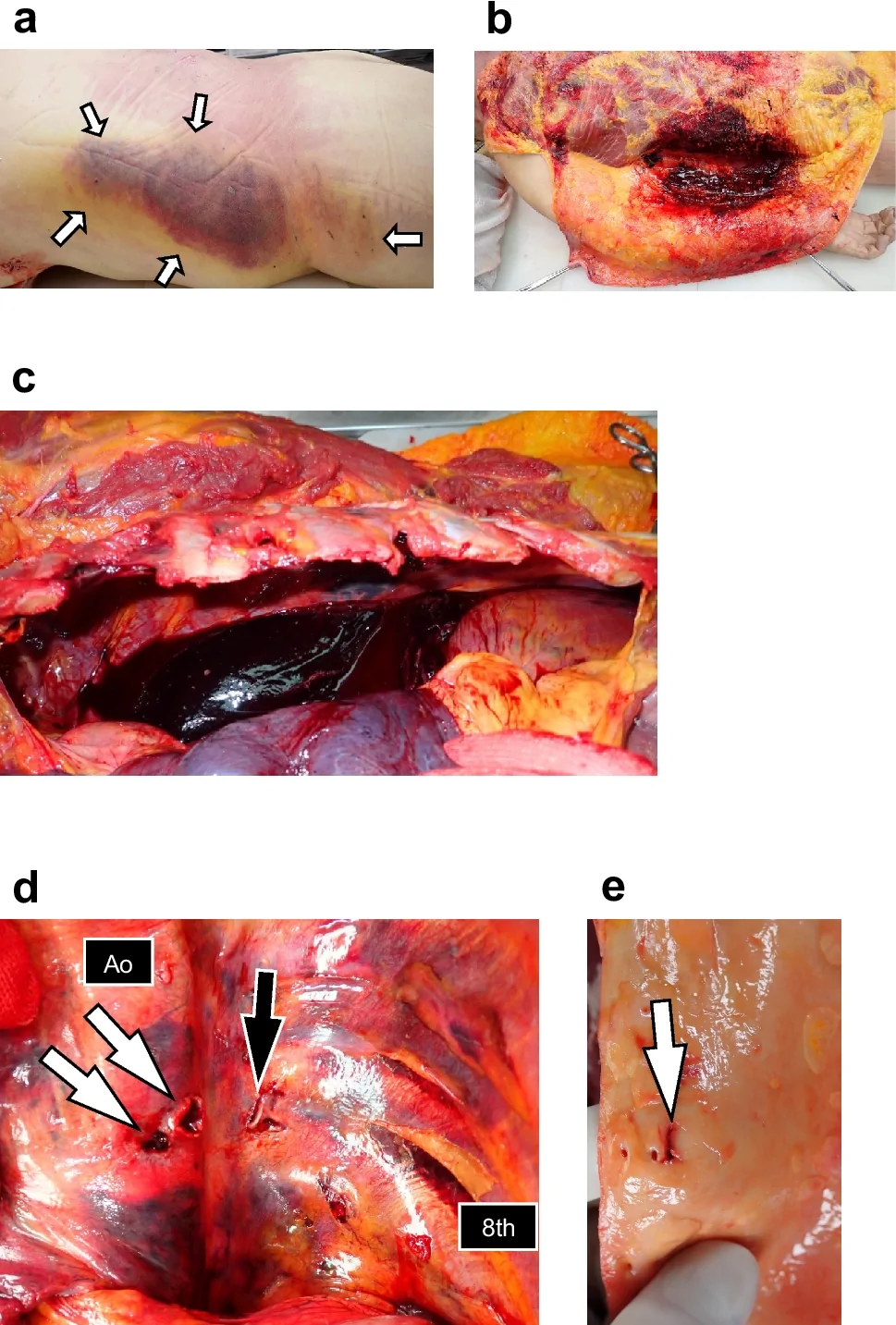
Autopsy photographs obtained nine days after the death. (**a**, **b**) A large subcutaneous hematoma and soft tissue hematomas on the left lower back. (**c**) Hemorrhagic pleural fluid in the left thoracic cavity. (**d**) Two aortic injuries (white arrows) are observed close to the edge of the left 8th rib fracture (black arrow), one of which is a full-thickness penetrating injury. (**e**) The intimal surface of the aortic wall. The penetrating injury was observed to be continuous from the adventitial surface

Autopsy revealed that the patient was 161 cm tall and weighed 61 kg. Externally, a large subcutaneous hematoma with multiple soft tissue hematomas on the left lower back was observed, consistent with the presumed impact site (Fig. 2a,b). Deep dissection revealed fractures of the left 8th–12th ribs near the costotransverse joints and fractures of the L1–L4 vertebrae, which were considered the source of the retroperitoneal hematoma.

Hemorrhagic pleural fluid (400 mL) was observed in the left thoracic cavity (Fig. 2c). Exploration of the hemorrhage source revealed two injuries to the descending thoracic aortic wall (Fig. 2d,e): 1) full-thickness penetrating injury (1.1 × 0.2 cm) directly aligned with the sharp edge of the fractured left 8th rib, and partial-thickness injury without intimal disruption, associated with a small surrounding mural hematoma.

Splenic capsule wrinkling and pronounced demarcation between the renal cortex and medulla (“shock kidney”) indicated massive left thoracic hemorrhage. The retroperitoneal hematoma extended from the spleen to pelvis, without evidence of active bleeding. No underlying diseases accounting for the sudden death were observed.

### Histopathology

**Fig. 3:**
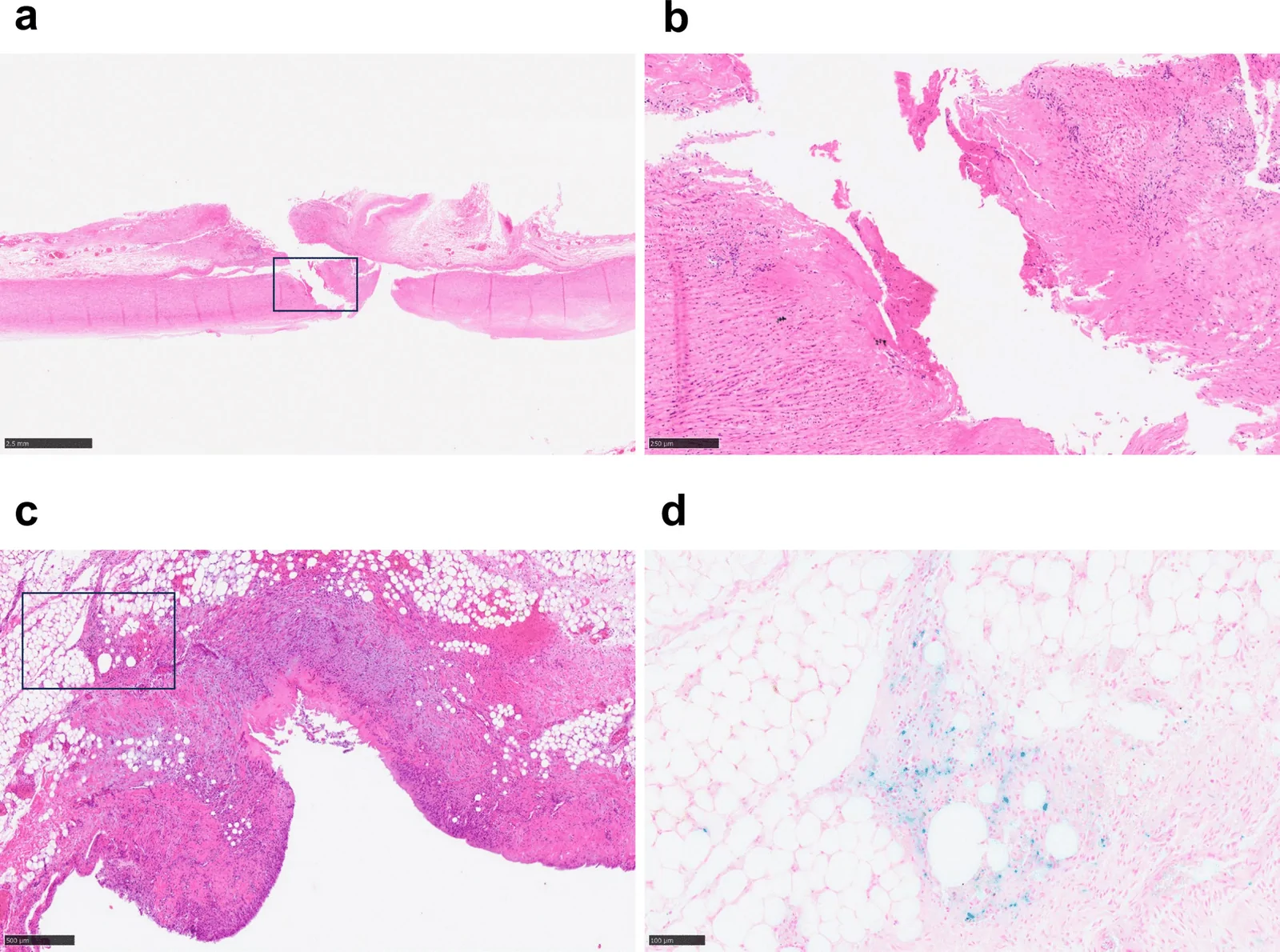
Histopathological images of the wall of the thoracic aorta. (**a**: × 1.25, **b**: × 10) Fresh fibrin deposition without obvious inflammatory cells at the site of full-thickness rupture. (**c**) Fibroblast proliferation in the adventitia adjacent to the rupture site. (**d**) Hemosiderin deposition in the adventitia. (**a**–**c**) Hematoxylin and eosin staining, (**d**) iron staining

Microscopic examination of the aorta revealed full-thickness rupture of the aortic wall. There was no obvious inflammatory cell infiltration at the rupture site, and fresh fibrin deposition was observed, indicating that the lethal perforation occurred shortly before his death. (Fig. 3a,b). Minimal intimal atherosclerotic change and preserved medial elastic fiber suggested that the rupture was not due to aortic wall vulnerability. In contrast, the adventitia adjacent to and surrounding the rupture exhibited fibroblast proliferation and hemosiderin deposition, showing repeated minor trauma or subclinical injury to the outer aortic wall several days before the terminal event (Fig. 3c,d).

The retroperitoneal hematoma contained focal areas of fibroblast proliferation and hemosiderin deposition, similar to the findings in the aortic adventitia. This indicates that these lesions began to form at approximately the same time. The brain exhibited a localized subarachnoid hemorrhage with hemosiderin-laden macrophages, but there were no significant parenchymal lesions. Other major organs, including the heart, lungs, liver, kidneys, spleen, pancreas, and adrenal glands, showed no acute pathological findings that could explain sudden death. No evidence of infection was observed. The thyroid gland showed only mild focal lymphocytic infiltration, compatible with minimal chronic thyroiditis.

## Discussion

Delayed aortic injury from posterior rib fractures is an uncommon but well-recognized complication of blunt thoracic trauma [4, 6–9, 11–13, 15]. Posterior fractures of the left lower ribs, particularly the 4th–10th ribs, carry the greatest risk owing to their anatomical proximity to the descending aorta. Instability caused by the multiple rib fractures and the mechanical forces of the surrounding muscles (e.g., the erector spinae), which can displace sharp bone fragments, increases aortic injury risk [8, 9, 11–13]. In our case, although the fractured 8th rib was anatomically close to the descending aorta, the initial CT scan suggested no evidence of aortic injury. Therefore, clinical attention was primarily directed toward other major findings, including a large retroperitoneal hematoma and a suspected pseudoaneurysm. We reviewed the pre- and post-mortem CT images and found that the edge of the left 8th rib fracture was adjacent to the descending aorta. Post-mortem CT revealed irregularity of the aortic wall at the level of the fracture, accompanied by massive pleural fluid in the left thoracic cavity. We concluded that the cause of death was exsanguination due to a penetrating aortic injury attributed to repetitive mechanical irritation by a posterior rib fracture; this was supported by histopathological findings. Moreover, fibroblast proliferation in the adventitia around the rupture site indicated repeated minor trauma to the aortic wall over several days, suggesting that the fatal rupture was not a single instantaneous event but rather the culmination of progressive injury.

The progressive left pleural effusion possibly resulted from hemorrhage around the fractured ribs and non-hemorrhagic pleural effusion. While histopathology suggested that the full-thickness aortic rupture probably occurred near the time of death, intermittent minor leakage from the aortic wall due to repetitive contact was also present; these processes coexisted. However, during follow-up, only chest radiographs were performed, and no detailed evaluation was conducted until the patient’s sudden deterioration. Therefore, the pleural fluid accumulation rate before death remains unclear.

Regarding the retroperitoneal hematoma, hemorrhage from local vessels associated with lumbar vertebral fractures was considered the plausible source, evidenced by the transient appearance and resolution of the pseudoaneurysm along with hematoma volume changes. In our case, the aortic wall had likely already been weakened by cumulative microtrauma; therefore, even a trivial movement, namely, slight upper body elevation during a meal, could precipitate fatal rupture. This finding underscores that minimal mechanical stress can trigger catastrophic failure following repetitive injury. Previous reports have described a variable latency period (hours to > 10 days), during which patients may remain clinically stable before sudden circulatory collapse due to massive hemothorax [6, 7, 9, 12]. A summary of these previously published cases, including the characteristics of their latency periods and outcomes, is presented in Table 1 [6–31]. Traumatic aortic injury is associated with high mortality, with a substantial proportion of patients dying before hospital arrival [32–37]. Therefore, early detection and prompt intervention are crucial [10, 15, 30, 31, 38–40].

**Table 1:** Summary of previously reported cases of delayed thoracic aortic injury caused by rib fractures

Authors, Year	Sex	Age (y)	Time from injury to onset	Left-sided injured ribs	Threatening ribs	Triggering event	Outcome
Matsumura et al., 1989 [16]	Male	72	2 days	5th—11th	7th	N/A	Alive
Kigawa et al., 1992 [10]	Male	37	10 days	Multiple rib fractures	8th	Changing position	Alive
Marco et al., 1997 [17]	Male	36	3 days	Multiple rib fractures	6th	Spontaneous pseudoaneurysm rupture	Alive
Tsai et al. 1999 [11]	Female	65	Day of injury	4th—8th	Not reported	Frequent bed transfer and mobilization during tests	Dead
Iyoda et al., 2003 [18]	Male	66	15 days	Multiple rib fractures	9th	Suspected body-position change	Alive
Ashrafian et al., 2005 [12]	Male	52	11 days	Left multiple rib fractures	Not reported	Rolling and chest physiotherapy	Alive
Kabiri et al., 2007 [19]	Female	24	Day of injury	Multiple rib fractures	9th—10th	N/A	Alive
Yanagawa et al., 2008 [20]	Male	57	5 days	6th—10th	7th	Changing position	Alive
Bruno et al., 2009 [21]	Male	87	6 days	8th—9th	8th	Spontaneous clot dislodgement	Alive
Faneyte et al., 2009 [22]	Male	56	Day of injury	Multiple rib fractures	Not reported	N/A	Alive
Kitamura et al., 2012 [23]	Male	63	2 days	5th—11th	7th	Spontaneous clot dislodgement	Alive
Boyles et al., 2013 [9]	Male	50	2 days	1st—11th	7th—8th	N/A	Dead
Kano et al., 2014 [24]	Female	55	Day of injury	3rd—8th	7th	N/A	Dead
Morimoto et al., 2014 [25]	Male	62	2 days	9th	9th	N/A	Alive
Park et al., 2014 [27]	Male	66	4 days	3rd—12th	7th	N/A	Alive
Na 2017 [14]	Male	49	Day of injury	1st—12th	4th	N/A	Dead
Pagliariccio et al., 2018 [26]	Female	89	Day of injury	9th	9th	N/A	Alive
Xie et al., 2019 [13] (Case 1)	Female	34	13 days	4th—8th	4th—6th	Surgical position for amputation	Alive
(Case 2)	Male	66	11 days	8th—11th	10th	N/A	Alive
Kai et al., 2021 [15]	Male	72	1 day	9th—11th	9th	Changing position	Alive
Lim et al., 2022 [28]	Female	74	1 day	4th—11th	7th	Shoulder dislocation and reduction	Dead
Masuda et al., 2022 [29]	Female	78	22 days	9th—11th	9th	N/A	Dead
Wang et al., 2022 [8] (Case 1)	Male	62	4 days	3rd—9th	7th	NA	Alive
(Case 2)	Male	63	3 days	3rd—11th	6th	N/A	Alive
(Case 3)	Male	51	2 days	2nd—11th	8th	N/A	Alive
(Case 4)	Male	58	3 days	2nd—10th	8th	N/A	Alive
(Case 5)	Male	43	3 days	2nd—11th	8th	N/A	Alive
(Case 6)	Female	54	1 day	3rd—10th	5th—8th	N/A	Alive
(Case 7)	Female	64	2 days	1st—11th	7th	N/A	Alive
Dakic et al., 2023 [30]	Male	60s	Day of injury	4th—8th	4th—5th	N/A	Alive
Maier et al., 2024 [31]	Male	49	Day of injury	5th—7th	5th	N/A	Alive
Nakano et al., 2025 [6]	Female	70s	9 days	9th—11th	10th	Changing position	Alive
Travers et al., 2025 [7]	Female	84	2 days	9th—11th	9th—11th	Spontaneous displacement or second fall	Alive
Our case	Male	62	10 days	8th—11th	8th	Changing position	Dead

**Abbreviation:** *N/A* not available

Similar clinical trajectories have been reported in other types of skeletal trauma. In a case of pelvic fracture, a patient who was initially hemodynamically stable experienced sudden arterial hemorrhage 72 h after admission [41]. Similarly, Lei et al. reported a case of a T10 vertebral fracture where the aortic injury was not detected on initial CT scans and remained undiagnosed until the following day after transfer [42]. These reports demonstrated that major vascular complications can remain occult after a latent period following the initial trauma, even when the patient appears clinically stable and initial imaging is inconclusive, particularly when clinical findings can be accounted for by the concurrent skeletal injuries.

Aortic injury is mainly managed through endovascular repair and open thoracotomy. Endovascular repair is currently favored in many cases [26, 34, 36], although open repair remains necessary depending on anatomical considerations [43]. However, in cases of rib fracture-associated aortic injury, clinical management remains unstandardized. Moreover, surgical stabilization or resection of the fractured ribs has been proposed as a preventive strategy [38–40]. Although no formal guidelines exist, there is a consensus emphasizing early recognition and timely intervention. Wang et al. proposed preventive surgical intervention criteria for patients with bicritical and severe posterior fractures of the left 4th–10th ribs accompanied by ≥ 4 fractured ribs, multiple fractures in a single rib, or fracture ends located within the lateral erector spinae muscles [8].

Clinically, this case highlights that early recognition of aortic injury risk based on anatomical proximity and fracture configuration might have influenced clinical decision-making, including risk communication with the patient and consideration of preventive strategies. Surgical rib stabilization (open reduction and internal fixation) has increasingly been adopted, particularly in cases of flail chest, to improve respiratory function. Furthermore, additional prophylactic measures, including preemptive endovascular stent-graft placement before rib fixation, may be performed, although whether they should be routinely performed remains controversial. This approach might also have been considered a potential option in selected high-risk scenarios, such as in our case. However, further case accumulation is warranted to establish the definitive role of these preventative strategies.

Forensically, this case underscores the importance of correlating radiological, clinical, and pathological findings when assessing delayed traumatic complications. The organized adventitial fibrosis observed at the rupture site provided objective evidence of repeated premortem mechanical stress, supporting the diagnosis of delayed traumatic rupture rather than spontaneous aortic disease. Overall, through integrated analysis of ante- and post-mortem imaging and detailed pathological examination, we demonstrated that even minor body movements can precipitate fatal aortic injury with unstable rib fractures.

## Conclusion

Delayed aortic injury secondary to posterior rib fractures is a well-documented but often underrecognized complication of blunt thoracic trauma. This case demonstrates that fatal aortic rupture can occur after minor body movements when the aortic wall has been progressively weakened by repetitive mechanical irritation. This case underscores the need for heightened vigilance and proactive reassessment when rib fractures are close to the aorta. From a medicolegal perspective, this case illustrates the importance of recognizing both overt injuries and foreseeable lethal complications inherent in specific trauma mechanisms.

## Data Availability

Data related to this study are available from the corresponding author upon reasonable request.
